# Electrospun Nanofiber Membrane: An Efficient and Environmentally Friendly Material for the Removal of Metals and Dyes

**DOI:** 10.3390/molecules28083288

**Published:** 2023-04-07

**Authors:** Li Li, Wei Guo, Shenggui Zhang, Ruibin Guo, Li Zhang

**Affiliations:** 1 College of Science, Gansu Agricultural University, Lanzhou 730070, China; 2 Research Center of Gansu Military and Civilian Integration Advanced Structural Materials, Key Laboratory of Eco-Environment-Related Polymer Materials, Ministry of Education of China, Key Laboratory of Polymer Materials of Gansu Province, College of Chemistry and Chemical Engineering, Northwest Normal University, Lanzhou 730070, China; 3 College of Food Science and Engineering, Gansu Agricultural University, Lanzhou 730070, China; 4 State Key Laboratory of Cryospheric Science, Northwest Institute of Eco-Environment and Resources, Chinese Academy of Sciences, Lanzhou 730070, China

**Keywords:** electrospinning, nanofiber membrane, metals and dyes

## Abstract

With the rapid development of nanotechnology, electrospun nanofiber membranes (ENM) application and preparation methods have attracted attention. With many advantages such as high specific surface area, obvious interconnected structure, and high porosity, ENM has been widely used in many fields, especially in water treatment, with more advantages. ENM solves the shortcomings of traditional means, such as low efficiency, high energy consumption, and difficulty in recycling, and it is suitable for recycling and treatment of industrial wastewater. This review begins with a description of electrospinning technology, describing the structure, preparation methods, and factors of common ENMs. At the same time, the removal of heavy metal ions and dyes by ENMs is introduced. The mechanism of ENM adsorption on heavy metal ions and dyes is chelation or electrostatic attraction, which has excellent adsorption and filtration ability for heavy metal ions and dyes, and the adsorption capacity of ENMs for heavy metal ions and dyes can be improved by increasing the metal chelation sites. Therefore, this technology and mechanism can be exploited to develop new, better, and more effective separation methods for the removal of harmful pollutants to cope with the gradually increasing water scarcity and pollution. Finally, it is hoped that this review will provide some guidance and direction for research on wastewater treatment and industrial production.

## 1. Introduction

In recent decades, mining, agriculture, chemical, and commodity production have severely degraded water resources. Water pollution comes mainly from the discharge of wastewater containing heavy metal ions from the mining and smelting industries, mainly from the extensive use of pesticides in industry and from the discharge of wastewater. Irreversible damage is caused to the environment. Heavy metal ions have become one of the many serious environmental problems. Heavy metal ions are commonly used in metal, leather tanning, metal manufacturing, and nuclear power plants [1,2,3,4,5]. Heavy metals are very difficult to biodegrade; instead, they can be concentrated up to several thousand times in the amplification of the food chain and end up in the human body. Heavy metals can interact strongly with proteins and enzymes in the human body, rendering them inactive. They can also accumulate in the body’s organs, leading to chronic poisoning. Mercury, arsenic, cadmium, hexavalent chromium (Cr), and other heavy metal ions can cause teratogenicity and are highly toxic. Even at low doses, they can pose a serious risk to human health because they accumulate in the body [6,7,8,9].

Dyes have been used for many years in the printing, leather, textile, and food industries [10,11,12,13,14]. Both natural and synthetic dyes can cause serious environmental pollution, and dye wastewater is a major pathway for many water pollution sources. Dyes in wastewater have complex and stable structures, large molecular weights, low biodegradability, and even low concentrations of dyes can have a significant impact on human health. Therefore, the removal of free dyes before the wastewater is discharged into natural waters is important for environmental protection [15,16]. Most dyes are highly toxic and readily soluble in water. Among them, aromatic amines, which are dye intermediates, have been classified as suspected carcinogens by many local authorities and are considered to be the most likely carcinogens in nature.

In the past, various methods have been used to remove heavy metal ions and organic dyes from water, such as membrane separation, reduction precipitation, ion exchange, coagulation–flocculation, adsorption, etc. [17,18,19,20]. Most removal methods are subject to stringent operating conditions and may also produce secondary contaminants with low efficiency. Among these technologies, adsorption is a superior physical and chemical method for the removal of organic dyes and heavy metal ions. To ensure the better adsorption of heavy metals and dyes, adsorbents should be prepared with a large surface area, high reusability, and strong interaction with heavy metal ions and organic dyes. For example, metal–organic skeleton compounds (MOFs) have been applied in adsorption due to their porosity properties, tunable structure, large specific surface area, and easy modification. Madhu N. Nimbalkar synthesized UiO-66-free carboxylic metal–organic skeleton using 1,2,4,5 phenyl tetracarboxylic acid, which has a high adsorption capacity for Pb^2+^ ions [21]. At the same time, graphene-based compounds have also been widely used in wastewater treatment. For example, regarding a GO/PVDF nanofiber membrane prepared by Fang-fang Ma, the adsorption capacity of MB was 621.1 mg g^−1^ [22]. At the same time, CNT [23] and MXene [24] have also been widely used in wastewater treatment. However, the preparation process of the above-mentioned powder adsorbents is complicated, the absorbents are difficult to recover, and the process involves easy to produce by-products, which cause secondary pollution. ENM has the advantages of a large specific surface area, high porosity, easy preparation, and no secondary contamination, which makes it very suitable for wastewater treatment [25].

Electrospinning technology mainly uses high power to stretch the polymer solution into a Taylor cone, and when the electric field force overcomes the surface tension, a charged jet can be injected from the top of the cone [26], which due to solvent evaporation and instability of the charged jet allows the solid nanofibers to be deposited randomly on the collector, forming a non-woven nanofiber membrane. Nanofiber membranes have the advantages of large volume ratios and ideal porosity and can be used for energy storage [27], water treatment [28], air filtration [29], ion exchange membranes [30], etc. The electrospinning technique has been widely used for the preparation of nanofiber membranes due to its advantages of mass producibility, speed, and simplicity [31]. Nanofiber membranes are now widely used for the treatment of pollutants in aqueous solutions due to their high specific surface area, easy surface modification, and porous structure [32,33]. This review references the work of researchers over the past five years, and a small amount of classic literature over a slightly longer period, on the removal of metals and dyes from wastewater by ENMs, which focuses on the structure, properties, influencing factors, and some of the working mechanisms of ENMs. The references are from a wide range of sources but mainly focus on nanofiber membrane materials and industrial applications and hopefully will provide some meaningful ideas for colleagues.

## 2. Preparation and Structure of Nanofiber Membrane

### 2.1. Electrospinning Technology

**Figure 1:**
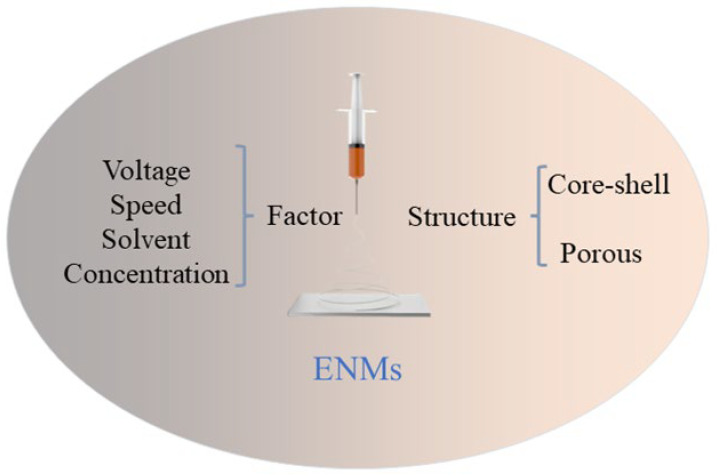
Preparation and structure of ENMs.

ENMs are used to produce fibrous membranes with quasi-3D diameters in the sub-micron or nano dimensions. Electrospinning is an aspecific type of fiber fabrication process that allows a polymer solution or melt to be jet-spun in a strong electric field, where the feedstock is transferred through the tip of a needle to a conical spherical tip (“Taylor cone”) and from the top of the cone, polymer filaments with nanoscale dimensions are produced [34,35], resulting in nanofiber membranes. The single-jet electrospinning device, often used in the laboratory, is shown in Figure 1 and consists of four components: a high-voltage power supply, a rotating liquid thruster, an ejector, and a grounded metal collector to collect the nanofibers [36,37]. The high-voltage power supply is available in the range of 0–50 kV, and the injector is primarily used to control the rate of injection of the rotating solution. In particular, small droplets can form on the spinnerets due to the polymer’s surface tension, and when the droplets are charged under the high voltage, the applied electric field is increased, and the counteracting electric field forces are correspondingly increased. When the electric field strength increases to a certain point, the electric field force is greater than the extension of the surface tension, and the deflected tip forms a Taylor cone and flies from the spinneret to the receiving hole as a jet stream [38].

### 2.2. Factors Affecting Electrospinning

**Figure 2:**
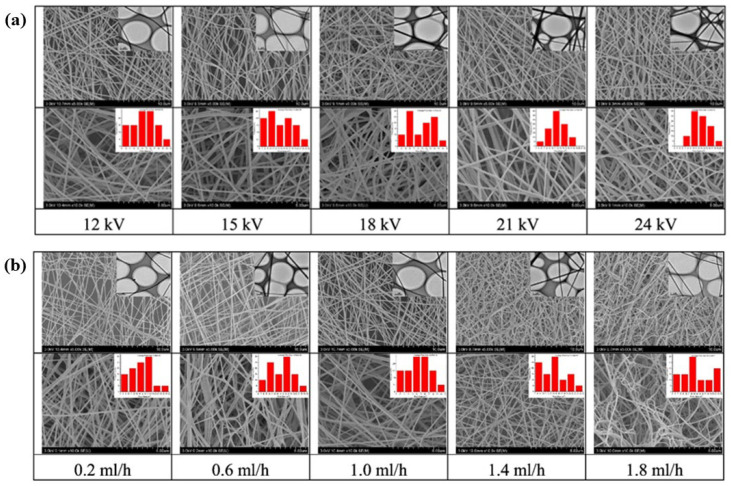
(**a**) SEM and TEM images of NFM at different voltages (the inset is the hole diameter distribution map). (**b**) SEM and TEM images of ENM at different flow rates (the inset is the pore diameter distribution map). Reprinted from ref. [39].

Several factors can affect the outcome of electrospinning in the production of ENMs. The first is the effect caused by voltage, as high voltage helps to stretch the fiber and increase the surface charge density of the jet and reduce the fiber diameter; however, voltages that are too high can lead to gelation, which can reduce the efficiency of fiber collection [26]. Voltage also affects the diameter of the ENM, and according to the literature [39], as shown in Figure 2a, as the applied voltage increases, the pore diameter of the ENM first decreases and then increases. This means that if the applied voltage is too high or too low, it will affect the increase in the pore diameter of the obtained ENM and the stability of the injection. Secondly, the injection speed is also an important parameter that affects the electrospinning process. If the speed is too slow, the electrospinning time will be prolonged, but if the injection speed is too fast, it will lead to the formation of larger fiber diameters or large droplets in the nanofiber film, which will hinder the effectiveness of electrospinning. As shown in Figure 2b, the pore size of the ENM gradually decreases when the flow rate is increased. In addition, when the flow rate is too high or too low, the pore size distribution is less uniform, and at the same time, beads cannot be formed when the flow rate is too high. 

**Figure 3:**
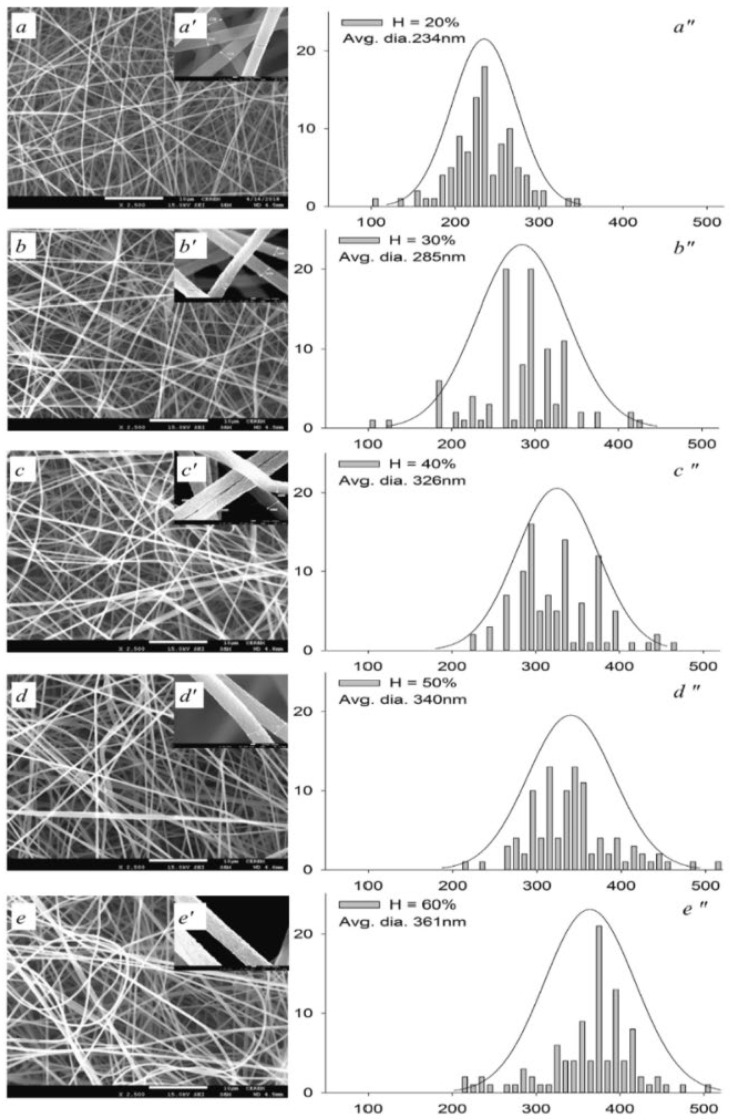
The effect of the distance from the tip to the collector on the ENM (**a**–**e**) at distances of 100 mm, 150 mm, 200 mm, 250 mm, and 300 mm, respectively. The bar scale in (**a**–**e**) is 10 μm and in (**a′**–**e′**) is 100 nm. Reprinted with permission from ref. [40]. Copyright 2019 Elsevier.

Again, the distance between the needle and the receiver also affects electrospinning. If the distance between them is too short, solvent evaporation will be adversely affected, leading to bonding between fibers, increasing the fiber diameter, and reducing the fiber performance. If the spinning distance is too long, the spinning nozzle will gain more opportunities to split, and the diameter of the fibers obtained will be greatly reduced. As shown in Figure 3, when the distance is too short (100 mm), the fibers will melt, possibly due to a lack of solidification time before the fibers are ejected onto the collector [40], and when the distance increases, the diameter of the ENM increases and the fiber morphology shows a small non-beaded fiber mat. If the distance is too long (300 mm), the diameter increases due to the weak electrostatic force, and beads may form, resulting in the solvent being trapped within the fibers [41]. At the same time, relative humidity and temperature also affect electrospinning [42]. Lower relative humidity will accelerate the evaporation rate of the solvent in the jet, which helps to form finer fibers. The temperature has two opposite effects on average fiber diameter. Higher temperatures accelerate the evaporation rate of the solvent. Lower temperatures will reduce the viscosity of the solution and promote the formation of finer fibers. Finally, electrospinning technology is also affected by the concentration of the solution, as too high a concentration of the solution will prevent jetting, but too low a concentration of the solution will eject small droplets, and electrospinning can only be performed when the concentration of the polymer is within a suitable range.

### 2.3. Nanofiber Membrane Structure

**Figure 4:**
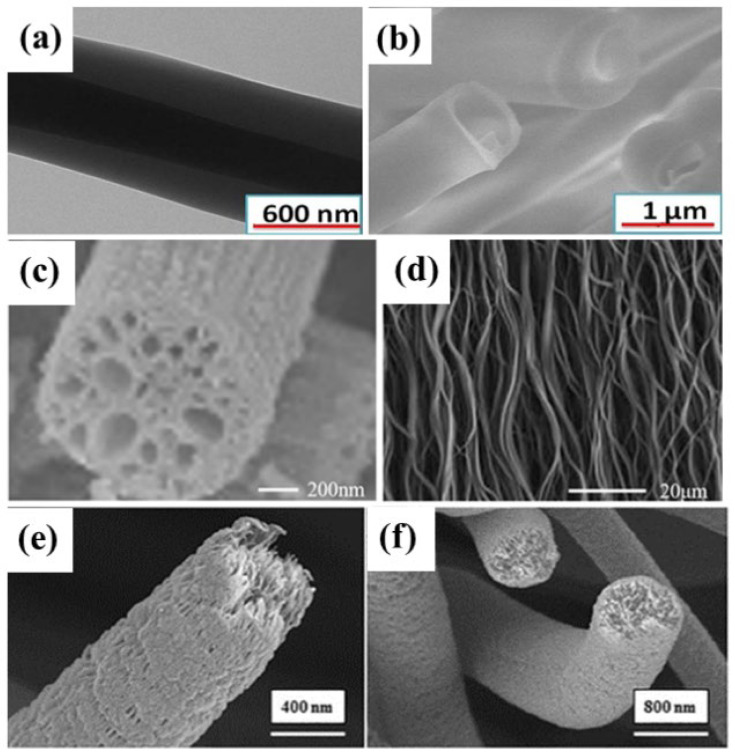
SEM images of NEM with different morphologies, (**a**) Core-shell structure; (**b**) Hollow structure; Reprinted with permission from ref. [44] Copyright 2017 Elsevier. (**c**) Porous structure; (**d**) Aligned structure; Reprinted with permission from ref. [34] Copyright 2020 Elsevier; (**e**,**f**) porous PAN micro/nanofiber membrane Reprinted with permission from ref. [43]. Copyright 2018 Elsevier.

In electrostatic technology, the structure of the ENM can be transformed by adjusting the voltage parameters or other conditions to meet the requirements of the experiment. In this section, several methods of preparing nanofibers with complex internal structures are presented [43,44,45,46,47,48,49]. Figure 4 is a schematic diagram of the structure of a common nanofiber membrane.

#### 2.3.1. Core–Shell Structure Electrospray Nanofiber Membrane

**Figure 5:**
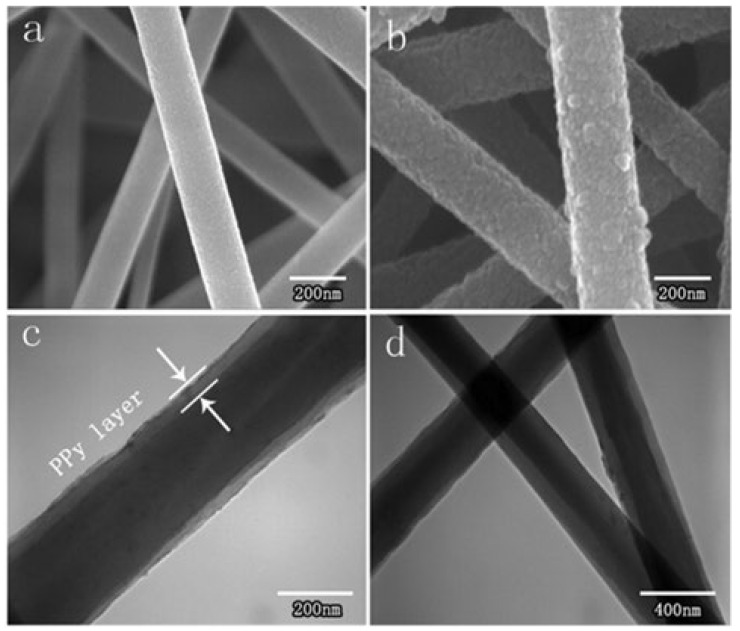
(**a**) Pure PAN nanofiber; (**b**) PAN/Ppy nanofiber; (**c**) PAN/Ppy nanofiber; (**d**) SEM and TEM image of PAN/Ppy nanofiber membrane. Reprinted with permission from ref. [50]. Copyright 2013 Elsevier.

Compared with ordinary electrospinning nanofibers, nanofibers with “core–shell” structures can synergistically exploit the advantages of both “core” and “shell” structures and exhibit better performance. According to the report by Jian Qiang Wang [50], the specific method for the preparation of a polyacrylonitrile/polypyrrole (PAN/PPy) core–shell structure is provided. Figure 5 is an SEM photograph of the prepared ENM, with Ppy(polypyrrole) well deposited on the front side of the ENM. Moreover, Xue Wu Huang reported [51] that flexible ultra-thin “core–shell” structures, and hydrophilic and conductive polymer nanofiber composites with unique PDA/ACNT “shell” and polymer nanofiber “core” structures were prepared by decorating polyurethane (PU) nanofibers with acidified carbon nanotubes (ACNTs) and then modified with polydopamine (PDA), which showed great potential in water purification. As reported by Fang-fang Ma [52], Ppy particles were deposited on polyvinylidene fluoride (PVDF) nanofibers using polydopamine (PDA) to produce new composite nanofibers with a similar structure of the “core–shell”. The morphological characteristics confirmed the electrospun PVDF/PDA nanofibers were uniformly encapsulated by Ppy particles, forming a typical core–shell structure. 

In the core–shell structure of an ENM prepared by electrospinning, compounds such as PAN, PVDF, etc., are modified or coated on the surface of the ENM to form a functionalized nanofiber membrane. Under normal circumstances, the core part is mainly the supporting part, while the shell part is the functionalized part, and in most cases, the function of the ENM is added or changed, mainly by modifying the “shell”. In the ENM used for water purification (WP), the properties of the “shell” can be modified to improve the purification function of the ENM, providing a new idea for future wastewater treatment work, but in this regard, attention must be paid to the effect of non-uniform deposition of compounds on the surface of the nanofiber membrane.

#### 2.3.2. Porous Nanofiber Membrane

**Figure 6:**
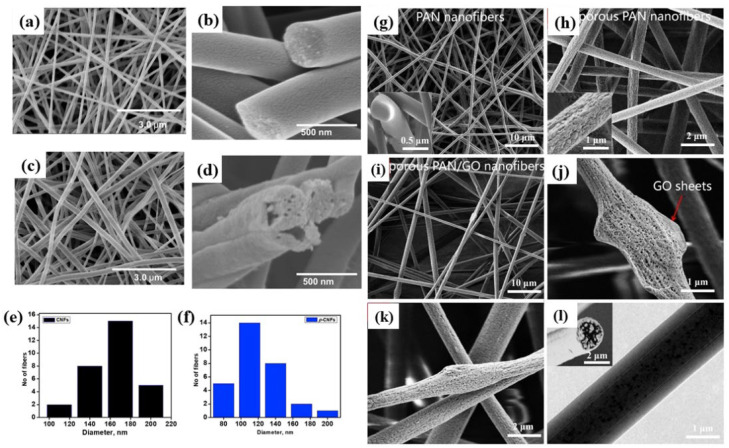
(**a**,**b**) SEM photos of CNF; (**c**,**d**) SEM photos of p-CNF; (**e**,**f**) the diameter distribution diagrams of CNF and p-CNF, respectively. Reprinted with permission from ref. [53]. Copyright 2019 Elsevier; (**g**) PAN; (**h**) porous PAN; (**i**) SEM and TEM images of porous PAN/GO nanofibers; (**i**–**l**) porous structure on the shell and core of porous PAN/GO nanofibers (inset: cross section membrane performance). Reprinted with permission from ref. [54]. Copyright 2020 Elsevier.

According to a report by Faraz Khan Mahar et al. [53], PAN/poly(methyl methacrylate) (PMMA) nanofiber membranes were first prepared and then carbonized to convert the obtained nanofibers into p-CNF, which was then prepared to obtain porous nanofiber membranes. On the other hand, Zhang-Qi Feng et al. prepared porous polyacrylonitrile/graphene oxide nanofiber membranes [54] by adding PAN/GO solution to the N, N-dimethylformamide (DMF)/H_2_O solvent mixture to prepare porous polyacrylonitrile (PAN)/graphene oxide (GO) ENM by a simple one-step electrospinning method, the SEM of which is shown in Figure 6. From the current publicly reported research work, the prepared porous ENM has the following point: (1) the porous exterior can increase the specific surface area of the ENM, which can further improve the adsorption capacity of the ENM; (2) more importantly, the porous structure can well expose the compound loaded in the nanofiber to the water environment, so that the adsorption performance of the nanofiber membrane can be better reflected [55,56].

## 3. Applications of ENM Wastewater Treatment

A variety of ENMs are used in wastewater treatment. For example, nanofiltration membranes [57], nanofiltration ultrafiltration membranes [58], and microfiltration membranes [59] are used to remove wastewater. However, the above methods are accompanied by membrane pollution and high energy consumption. Nanofiber membranes as adsorbents have the advantages of no energy consumption and less membrane pollution; therefore, they are used for wastewater treatment. Here, two-dimensional materials (GO, CNC, etc.) have hydroxyl, carboxyl, amino, and other functional groups, which have high adsorption performance for heavy metal ions and dyes. For example, Senelisile M. summarized the application of nanocellulose-based nanofiber membranes containing carbon nanomaterials for dye wastewater treatment. Because carbon nanomaterials have favorable physical and chemical properties, such as strength, stiffness, green color, renewability, and sustainability, they are doped in the nanofiber membranes to improve the adsorption capacity of the membranes. At the same time, MOF-modified membranes showed excellent selectivity and permeability and minimal structural defects on the matrix [60]. In addition, MOFs can be used as dopants in heavy metal adsorption because of their easier controllability of pore size and structure. In this review, two-dimensional materials are added to nanofiber membranes to increase the adsorption sites on the membrane surface and improve the removal ability of the membrane to heavy metal ions and dyes in wastewater.

### 3.1. Heavy Metal Ion Removal

#### 3.1.1. Heavy Metal Ion Adsorption 

**Figure 7:**
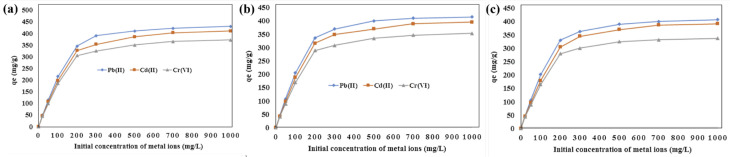
Effect of initial concentration of (**a**) Pb (II), (**b**) Cd (II), and (**c**) Cr (VI) ions on the adsorption of metal ions using PAN/chitosan/UiO-66-NH_2_ nanofibrous adsorbent. Reprinted with permission from ref. [64]. Copyright 2019 Elsevier.

It is well known that some specific functional groups can form stable chemical bonds with heavy metal ions for wastewater treatment [13,55,61,62,63]. Amine, hydroxyl, and carboxyl groups can form complexes with metal ions for the adsorption of metal ions. For example, Sana Jamshidifard et al. [64] investigated the incorporation of UiO-66-NH_2_ MOF into PAN/CS and successfully prepared nanofiber membranes. The membrane showed strong adsorption performance for Pb (II), Cd (II), and Cr (VI) reaching 441.2, 415.6, and 372.6 mg·g^−1^, respectively. As shown in Figure 7, good adsorption performance was maintained after five adsorption cycles. Based on the adsorption kinetics, it was shown that the complexation of amine plays a signification role in the metal ion adsorption process, indicating that chemisorption was the main factor in the adsorption of the ENM. According to E. Salehi [65], polyethylene glycol (PEG) and amino-modified multi-walled carbon nanotubes (MWCNT-NH_2_) are used to modify the thin adsorption of chitosan/polyvinyl alcohol (CS/PVA) membranes, where copper ions and amine groups can form complexes for copper ion adsorption. The idea of the study was consistent with that reported by Sheng Deng et al. [66], where the polyether-acrylamide-modified multi-walled carbon nanotubes were co-spun with polyacrylonitrile trimers to prepare a composite nanofiber membrane with excellent adsorption of Cu^2+^ and Pb^2+^. The main mechanism is that the PEI molecule attached to the MWCNT contains primary, secondary, and tertiary amine groups in a ratio of about 1:2:130, and the adsorption of metal ions is due to the action of the hydroxyl group and amine group on the nanocomposite membrane [67]. The ion exchange between the metal ions and the hydroxyl ions reacted on Co^2+^ sites. According to Shuping Wu et al. [68], CS/PVP/PVA nanofiber membranes were synthesized by electrospinning; the maximum monolayer adsorption capacities of Cu (II), Ni (II), Cd (II), and Pb (II) on nanofiber membranes are 34.79, 25.24, 18.07, and 16.05 mg g^−1^, respectively. This is mainly due to the formation of chemical bonds between adsorbent and adsorbent, i.e., the chelation of amino and hydroxyl groups with heavy metal ions. 

Chitosan (CS) has attracted great interest as an excellent adsorbent material with non-toxic and non-hazardous properties and has been extensively studied in the past. Chitosan (CS) is a natural polymer that can remove metal ions due to the presence of the amino group of the 2-amino-2-deoxy-d-glucose (glucosamine) unit [69,70]; mainly due to the possession of many amino groups and hydroxyl groups, functional groups can form stable complexes with heavy metal ions during adsorption [71,72]. According to Lei Li et al. [73], a layered CS nanofiber layer with an average diameter of 75 nm was successfully prepared by electrospinning using 5 wt % chitosan as the spinning solution, and then the nanofibers were crosslinked to remove Cr (VI) from water by static adsorption, and the maximum adsorption capacity of the nanofibers was 131.58 mg·g^−1^, which was twice that of chitosan powder. Lei Li also prepared a renewable spiral wound assembly of electrospun chitosan nanofiber membrane with a high affinity for the removal of Cr ions [74].

**Figure 8:**
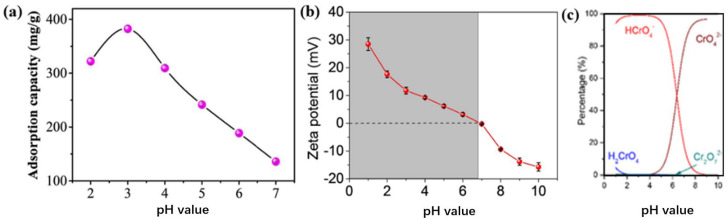
(**a**) The effect of the pH value of the Cr (VI) aqueous solution on the adsorption capacity of PAN/GO porous nanofibers; (**b**) zeta potential of porous PAN/GO nanofibers in aqueous solutions with different pH values; (**c**) The form of Cr (VI) at pH from stepwise from 1–10. Reprinted with permission from ref. [54]. Copyright 2020 Elsevier.

Electrostatic attraction is an important mechanism for the adsorption of heavy metal ions by NEM, and the pH of the solution in this process is the most important factor affecting the adsorption of heavy metal ions. The porous PAN/GO nanofibers with abundant nanopores [54,75] reached 382.5 ± 6.2 mg·g^−1^ for Cr (VI) adsorption, as shown in Figure 8a. The pH of the solution had a significant effect on the adsorption of ENM. The pH value of 3 reached the maximum adsorption, which gradually decreased as the pH value of the solution increased. The reasons for the change in adsorption capacity are as follows: the metal ion of Cr (VI) in the solution usually exists in three forms of chromate, namely, CrO_4_^2−^, Cr_2_O_7_^2−^, and HCrO_4_^−^. As shown in Figure 8c, HCrO_4_^−^ is the predominant form of Cr (VI) in an aqueous solution where the pH decreases and the deprotonated hydroxy nanofibers of PAN and GO sheets form a positively charged surface, which adsorbs the negatively charged HCrO_4_^−^ by electrostatic attraction. However, as the zeta potential of PAN/GO nanofibers decreases with increasing pH, the adsorption capacity also decreases, so it can be concluded that the negative surface of porous PAN/GO nanofibers decreases the adsorption capacity with increasing pH due to electrostatic forces. In addition, according to Palaniswamy Suresh Kumar et al. [15], most heavy metal ions (such as Pb^2+^, Cd^2+^, and Cu^2+^) are in the divalent state in water at low pH values. Due to the low pH value, the mixed CNFs/TiO_2_-PAN was positively charged, resulting in a strong electrostatic repulsion between metal ions. As the pH value increases, the electrostatic attraction gradually increases, and the adsorption of metal ions increases. The adsorption capacity of ENM for heavy metal ions is listed in Table 1, and it can be seen that the pH of the solution has a significant effect on the adsorption of the ENM. Meanwhile, the adsorption capacity of other adsorbents for heavy metal ions is listed, as shown in Table 2.

**Table 1:** The adsorption performance of other nanofiber adsorbents for heavy metal ions.

Nanofiber Adsorbent	Polymer	Surface Area (m^2^·g^−1^)	Heavy Metal Ions	Maximum Adsorption Capacity (mg·g^−1^)	pH	T (°C)	Sample Volume (mL)	Kinetics Model	Isotherms Model	Ref.
PDA/MnO_2_/PAN	PAN	66	Pb^2+^	185.19	6	25	/	Pseudo-second-order model	Langmuir isotherm model	[76]
PVA/PEI	PVA PEI	/	Cr^6+^	150	4	25	10	Pseudo-first-order model	Langmuir isotherm model	[77]
PAN/Fe_2_O_3_@Fe_2_O_3_	PAN	/	Pb^2+^	57	/	25	50	Pseudo-first-order model	Langmuir isotherm model	[78]
PAN-CNT/TiO_2_-NH_2_	PAN	/	Cr^6+^	714	2	20	100	Pseudo-first-order model	Freundlich isotherm model	[79]
PA6@Mg (OH)_2_	PA6	/	Cr^6+^	294.6	3	25	40	Pseudo-second-order model	Freundlich isotherm model	[80]
PVA@SiO_2_	PVA	370	Cu^2+^	489.12	6	25	100	/	Redlich-Peterson isotherm model	[81]
CS-PGMA-PEI	CS	/	Cr^6+^	138.96	2	25	10	Pseudo-second-order model	Langmuir isotherm model	[82]
EDTA-EDA-PAN	PAN	/	Cr^6+^	66	3	25	25	Pseudo-second-order	Freundlich isotherm model	[83]
m-PEI/PVDF	CCN	/	Cr^6+^	109	3	25	/	Pseudo-second-order	Freundlich isotherm model	[84]
HMO-PAN	PAN	/	Pb^2+^	194	7	25	20	Pseudo- second- order model	Freundlich isotherm model	[85]
CA/Fe_3_O_4_	CA	/	Pb^2+^	44	6	27	50	Pseudo- second- order model	Freundlich isotherm model	[86]
PA6/Fe_3_O_4_/o-MWCNTs	PA6	/	Pb^2+^	49	6	25	50	/	/	[87]
Lys-CNFs	CNFs	220	Pb^2+^	270	6	25	50	Pseudo-second-order model	Langmuir isotherm model	[88]
Thiol-functionalized cellulose	CS	/	Pb^2+^	22	4	25	50	Pseudo-second-order model	Langmuir isotherm model	[89]
CS-DTPA/PEO	CS	/	Pb^2+^	142	5	25	/	Pseudo-second-order model	Langmuir isotherm model	[90]
Hal/Fe_3_O_4_/PEO/CS	CS	38	Pb^2+^	67	7	25	/	Pseudo-second-order model	Langmuir isotherm model	[91]
Palygorskite/chitin	ChNFs	/	Pb^2+^	53.7	7	25	50	Pseudo-second-order model	Freundlich isotherm model	[92]
MgAl-EDTA-LDH@PAN	PAN	/	Cu^2+^	120.7	5	25	/	Pseudo-second-order model	Langmuir isotherm model	[93]

Note: “/” indicates no relevant data mentioned in the reference.

**Table 2:** The adsorption performance of other adsorbents for heavy metal ions.

Adsorbent	Surface Area	Adsorption of Metal Ions	Maximum Adsorption Capacity (mg·g^−1^)	pH	T (°C)	Sample Volume	Kinetics Model	Isotherms Model	Ref.
rGO/PEI-KOH	/	Cr^6+^	398	2	25	2	Pseudo-second-order model	Langmuir isotherm model	[76]
Fe_3_O_4_@Arg-PPy NC	22	Cr^6+^	322	2	25	/	Pseudo-second-order model	/	[77]
PPy-rGO/Fe_3_O_4_	80	Cr^6+^	226	3	30	40	Pseudo-second-order model	Langmuir isotherm model	[79]
Hierarchical MnO_2_ microspheres	252	Pb^2+^	139	3	30	100	/	Freundlich isotherm model	[82]
NTA-β-CD-CS	/	Hg^2+^	178.3	6	25	/	Pseudo-second-order	Langmuir isotherm model	[83]
C-phenylcalix pyrogallolarene	/	Cu^2+^	8	5	25	/	/	/	[94]
Sepiolite@polyethyleneimine/SA	/	Pb^2+^	1094	5.5	25	10	Pseudo-second-order model	Langmuir model	[95]
SA@PEI-CDs	/	Pb^2+^	380	4	25	10	Pseudo-second-order model	Freundlich isotherm model	[96]
PAAO cryogels	/	Pb^2+^	450	5	25	75	Pseudo-second-order model	Freundlich isotherm model	[97]
CS/PVP/PVA	2.12	Pb^2+^	16	/	25	/	Pseudo-second-order model	Langmuir isotherm model	[68]
Cellulose/chitosan/alginic acid hydrogels	/	Cu^2+^	760	/	25	/	Pseudo-first-order model	Freundlich isotherm mode	[98]
Nanocellulose/sodium alginate/carboxymethyl-chitosan	284	Pb^2+^	472	5	25	50	Pseudo-second-order model	Langmuir model	[99]

Note: “/” indicates no relevant data mentioned in the reference.

#### 3.1.2. Heavy Metal Ion Filtration

**Figure 9:**
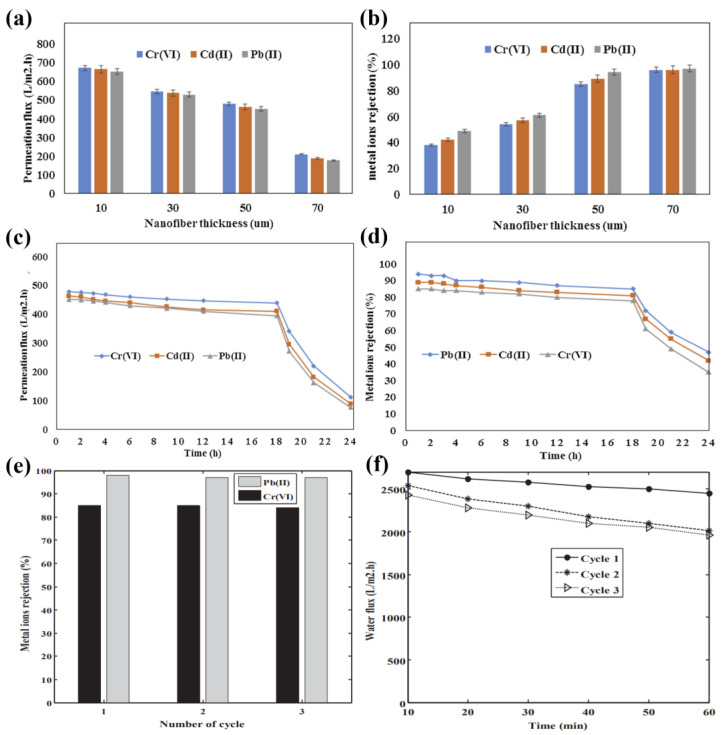
The effect of PAN/chitosan/UiO-66-NH_2_ nanofiber layer thickness on the removal of (**a**) flux and (**b**) metal ions using PVDF/PAN/chitosan/UiO-66NH_2_ nanofiber membrane, (**c**) permeation flux, and (**d**) removal efficiency of metal ions in the nanofiber membrane within 24 h. Reprinted with permission from ref. [64]. Copyright 2019 Elsevier; (**e**) regenerating metal on the PES/PVA/chitosan/A-Fe_3_O_4_-2 nanofiber membrane ion recovery rate; and (**f**) the influence of water flux. Reprinted with permission from ref. [100]. Copyright 2018 Elsevier.

Membrane adsorption is mainly divided into static adsorption and dynamic adsorption, where dynamic adsorption of membranes is also an important process of membrane adsorption. According to the reports of Sana Jamshidifard et al. [64], the effect of the PAN/chitosan/UiO-66-NH_2_ MOF nanofiber layer on flux and metal ion removal was investigated. As shown in Figure 9a, the permeate flux decreased, and the metal ion removal increased when the thickness of the nanofiber layer was increased. As the thickness of the ENM layer is increased, the permeate flux of metal ions also decreases, mainly because there is a less aqueous solution passing through the membrane, thus reducing the diffusion of the aqueous solution through the membrane. In addition, the increase in metal ion removal with increasing ENM is due to the chemical reaction between the functional groups of the ENM (such as amines) and metal ions, which increases the available active sites for metal ion adsorption and can adsorb large amounts of metals on the surface of the ENM. In the work shown in Figure 9c,d, the performance of the ENM to remove heavy metals was investigated for 24 h. The water flux and metal ion removal rate remained constant for 18 h, after which the number of metal ions and the removal rate decreased significantly with adsorption saturation. Shahnaz Koush kbaghi et al. [100] prepared aminated Fe_3_O_4_ nanoparticles filled with chitosan/PVA/PES bilayer nanofiber membranes used for the treatment and adsorption of Pb (II) and Cr (VI) ions from aqueous solutions, and the results showed that the maximum adsorption capacities for Cr (VI) and Pb (II) ions reached 509.7 and 525.8 mg·g^−1^, respectively. During the treatment process, increasing the thickness of the ENM layer resulted in a slight decrease in water flux and an increase in metal ion recovery. The removal effect of metal ions increased with the increase in the initial concentration of heavy metal ions, while the water flux did not change significantly. Figure 9e,f show the adsorption results of ENM during water treatment repeated for three cycles, which demonstrates the good utility of the synthesized ENM as an adsorbent membrane in industry. In addition, according to Mohammad Pishnamazi et al. [101], UiO-66-NH_2_ and ZIF-8 metal–organic framework nanoparticles (NMOFs) were incorporated into polyvinylidene fluoride (PVDF) for the separation of Cr(VI) ions by ultrafiltration membranes to investigate the synthesized PVDF/NMOFs monolayer and PVDF/chitosan/NMOFs bilayer nanofiber membranes. The results showed that the maximum flux of PVDF/chitosan nanofiber membrane containing 20 wt % UiO-66-NH_2_ was 470 L·m^−2^·h^−1^, and the Cr (VI) rejection rate was 95.6% and the adsorption capacity of the prepared nanofiber membrane for Cr(VI) was 602.3 mg·g^−1^ after five adsorption–desorption cycles. This indicates that PVDF/chitosan/UiO-66-NH_2_ nanofibers have a high potential in membrane separation and Cr (VI) ion adsorption. In conclusion, the thickness of the ENM nanofiber layer affects the adsorption and permeation flux of Cr (VI). As the thickness of the ENM layer increases, the chelating sites for metal ions increase, and a large number of metal ions can be removed simultaneously, and the removal rate of metal ions increases. 

#### 3.1.3. Summary of Heavy Metal Removal

The main reason for the adsorption of heavy metal ions by ENM is the chelation reaction between amino, hydroxyl, and other functional groups and metal ions. According to the result of adsorption kinetics, the most important reason influencing adsorption is chemisorption. According to this line of research, the adsorption capacity of ENM for heavy metal ions can be improved by increasing the metal chelating sites [102,103], which we believe can be implemented in the following ways: (1) doping more amino compounds into the electrospinning solution to improve the removal efficiency, but the amount of the doped compounds should be appropriate, as too much compound will lead to unsuccessful preparation of ENM or aggregation on ENM, thus reducing the adsorption efficiency of heavy metal ions; (2) surface chemical treatment of the surface of carbon nanotubes or graphene-based compounds. The surface chemical treatment uses chemical methods to introduce amino groups and other groups on the surface of the compound, mainly to increase the number of amino groups so that more amino groups react with metal ions and improve the removal efficiency of ENM. (3) ENM was prepared by using natural polymers, such as chitosan, which is an excellent environmentally friendly wastewater treatment material because it is non-toxic and non-polluting. (4) The prepared nanofiber membranes were chemically modified by introducing other groups and amino groups on their surface to promote the removal efficiency and hydrophilic appearance of ENM.

The adsorption of heavy metal ions by ENM is currently a new way to remove heavy metal ions from water, but the desorption process of heavy metal ions and its influencing factors are also worthy of great attention because it helps to realize the recycling of heavy metal ions. The desorption process of heavy metal ions by changing the temperature and adjusting the pH value of the solution provides a new idea for the recycling of heavy metal ions in the environment, where the question worthy of our consideration is how to improve the desorption recovery of nanofiber membranes. 

Overall, the preparation of ENM provides a technical option for the traditional heavy metal ion adsorption, but it must be pointed out that the preparation of ENM has a direct impact on the removal rate of metal ions, and researchers need to conduct in-depth studies to improve it. Certainly, dynamic adsorption is an important factor among many issues to be considered to improve the adsorption efficiency, the recyclability of ENM used as adsorbents, and the stability of ligands on the fiber membrane surface; the adsorption isotherms, kinetics, and thermodynamic properties of nanofibers also deserve further discussion.

### 3.2. Dye Removal

#### 3.2.1. Adsorption of Dyes

**Figure 10:**
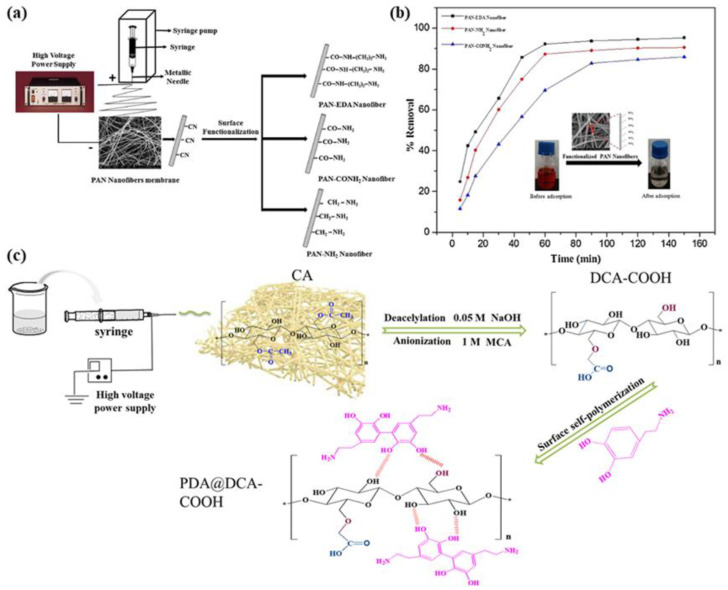
(**a**) Schematic diagram of functionalized PAN nanofibers; (**b**) adsorption of functionalized nanofiber membranes. Reprinted with permission from ref. [105]. Copyright 2018 Elsevier; (**c**) synthesis of PDA@DCA–COOH film. Reprinted with permission from ref. [12]. Copyright 2020 Elsevier.

Dyes and pigments are widely used in textile, leather, paper, cosmetics, plastics, printing, and other industries. These industries discharge non-biodegradable and complex organic pollutants into the water environment, posing a major threat to human health and aquatic organisms. Among them, Congo red (CR) is a synthetic antifouling agent, containing toxic aniline (aromatic amine) compounds that are suspected human carcinogens and mutagens [13,14,104]. As reported by S. Patel [105], polyacrylonitrile (PAN) nanofiber membranes synthesized by electrospinning were chemically modified with different amino (-NH_2_) functional groups on the surface and used as a novel nanosorbent for the removal of anionic Congo red dyes from aqueous media. They designed three chemical modus used to synthesize three different -NH_2_ functionalized nanofiber membranes, named PAN-NH_2_, PAN-CONH_2_, and PAN-EDA, whose structures are shown in Figure 10a, and the three different single bond NH_2_-functionalized PAN ENMs obtained were applied as adsorbents for the removal of toxic anionic CR dyes from aqueous media; the experimental results are shown in Figure 10b. It has been shown that the pH of the solution affects the removal of ENM, and at lower pH, the amino-functional groups present on the exterior of the functionalized nanofibers protonate, increasing the affinity for the removal of anionic CR dyes by creating a positively charged surface. However, at higher pH, the removal efficiency decreases due to the deprotonation of the amine group [106], which increases the negative charge on the surface of the functionalized nanofibers, causing electrostatic repulsion. According to F.F. Ma et al. [22], polyvinylidene fluoride (PVDF)/graphene oxide (GO) composite films were prepared by depositing graphene oxide on a polyvinylidene fluoride film using ultrasound, and the adsorption capacity of PVDF/GO composite films on methylene blue (MB) was investigated. The results showed that the adsorption ability of MB achieved 621.1 mg·g^−1^. As reported by F. F. Ma et al. [52], polydopamine (PDA) was used to modify electrospun PVDF nanofibers, and Ppy particles were deposited on the successfully modified nanofiber membranes to obtain composite nanofibers with a “core–shell” structure, and because it contains many nitrogen-containing groups, the surface roughness of the nanofibers was increased, and the hydrophilicity of the ENM appearance was greatly improved. They use Congo red (CR) and methylene blue (MB) to study the adsorption ability of ENM, and the results showed that the ENM had excellent adsorption capacity for both anionic and cationic dyes, with the maximum adsorption capacities of CR and MB being 384.6 and 370.4 mg·g^−1^, respectively. Z.M. Shourijeh prepared porous aminated PAN/PVDF composite nanofibers for the removal of Red 23 [107], and the dye removal capacity of the nanofibers was improved by extraction with NaHCO_3_ and modified diethylene triamine, and the removal of Red 23 was more than 95%. 

The removal efficiency of ENMs is closely related to the pH of the solution, mainly because pH affects the surface charge of the ENM, the degree of ionization of the dye, and the functional groups on the active site of the ENM [108]. Decreasing the pH increases the number of protons, which leads to the protonation of amino groups on the surface of the nanofiber membrane and the formation of NH_3_^+^ groups, and it improves the electrostatic attraction between the negatively charged dye and the positively charged ENM [109]. According to W.J. Chen et al. [12], a new economical multifunctional cellulose acetate was obtained by carboxymethylation, deacetylation, and a polydopamine (PDA) coating to remove both anionic and cationic dyes, and a schematic diagram of the preparation is shown in Figure 10c. The adsorption performance of the PDA@DCA-COOH ENM was determined to be 67.31 mg·g^−1^ for CR and 69.89 mg·g^−1^ for MB. The results show that the adsorption of the dyes by the ENM is related to the charge on the membrane surface and the pH of the solution. For MB, it is a typical cationic dye, and the outer layer of the ENM becomes negatively charged, increasing the electrostatic attraction between the ENM and MB, so the dye will be adsorbed under alkaline conditions, and therefore the amount of MB removed increases with the increase in pH. For Congo red, which is a common anionic dye, the adsorbent has a moderate removal rate for it under acidic conditions. At lower pH, the positively charged protonated amino groups (single bond NH_3_^+^) on PDA@DCA-COOH increase, and the electrostatic attraction between the adsorbent and CR increases. Therefore, PDA@DCA-COOH has excellent recovery and adsorption performance for both anionic and cationic dyes, and it is expected to be a new compatible and efficient adsorbent in wastewater treatment. 

We have compiled some research works on nanofiber membranes and listed the data for dye adsorption in wastewater treatment in Table 3 and Table 4 for comparison, which we hope will be a reference for readers.

**Table 3:** The adsorption performance of other nanofiber adsorbents for dyes.

Nanofiber Adsorbent	Polymer	Surface Area (m^2^·g^−1^)	Dyes	Maximum Adsorption Capacity (mg·g^−1^)	pH	T (°C)	Sample Volume	Kinetics Model	Isotherms Model	Ref.
PPy/PANi	PANi	/	CR	250	6	25	150	Pseudo-second-order model	Langmuir isotherm model	[110]
Gelatin/calcium Alginate	SA	/	MB	2046	4	25	25	Pseudo-second-order mode	Langmuir isotherm model	[16]
GO/CS	CS	/	MB	584	5	25	50	Pseudo-second-order model	Freundlich isotherm mode	[111]
Carboxylated Mn_2_O_3_	Carboxylated	148	MB	1175	9	25	400	Pseudo-second-order model	Langmuir isotherm model	[112]
Functionalized PAN	PAN	/	MG	200	8	25	/	Pseudo-second-order kinetic model.	Langmuir isotherm model	[113]
PVP/alumina	PVP	417	MO	351	5.5	25	30	Pseudo-second-order model	Langmuir isotherm model	[114]
PAN-CNT	PAN	/	MG	88	10	25	/	Pseudo-second-order model.	/	[115]
Sodium alginate/polyvinyl alcohol	PVA	/	MB	9	/	25	/	/	/	[116]
Vinyl-modified mesoporous poly(acrylic acid)/SiO_2_	PAA	523	MG	220		30	50	Pseudo-second-order model	Freundlich isotherm model	[117]
NiFe LDH/PAN/GO	PAN	/	RB	6.19	6	25	40	Pseudo-first-order model	Langmuir isotherm model	[42]
Mesoporous carbon	PVP	1642	MC	567	3	/	10	Pseudo-second-order kinetic model	Langmuir adsorption isotherm model	[118]
ZnO-HT-PAN_H	PAN	/	RB	267	/	25	15	Pseudo-first-order model	Langmuir isotherm model	[119]
PVDF/PDA	PVDF	/	MB	173	/	25	50	Pseudo-second-order model	Langmuir isotherm model	[120]

Note: “/” indicates no relevant data mentioned in the reference.

**Table 4:** The adsorption performance of other adsorbents for dyes.

Adsorbent	Surface Area (m^2^·g^−1^)	Dyes	Maximum Adsorption Capacity (m^2^·g^−1^)	pH	T (°C)	Sample Volume	Kinetics Model	Isotherms Model	Ref.
NTA-β-CD-CS	/	MB	162	6	25	25	Pseudo-second-order mode	Langmuir isotherm model	[89]
T-QT/CS	68.4	MB	917	9	25	/	Pseudo-second-order model	Langmuir isotherm model	[121]
CS_2_/CMC_2_-PEG	/	CR	1053	/	25	20	Pseudo-second-order model	Freundlich isotherm model	[122]
Pectin/graphene oxide aerogel	/	RhB	419	/	25	100	Pseudo-first-order model	Freundlich isotherm model	[123]
Benzenesulfonyl hydrazone modified guar gum	19	CR	1065	10	25	10	Pseudo-second-order model	Langmuir isotherm model	[124]
Barberry stem powder	/	RR 195	27	/	/	/	Pseudo-first-order model	Langmuir and Freundlich isotherms model	[125]
α-Fe_2_O_3_ nanoparticles	165	RR 195	20	/	/	/	Pseudo-second-order model	Langmuir isotherm model	[126]
Ca-alginate/citric acid (CA)-sawdust/UiO-66-NH_2_ hydrogel beads	15	MB	25	6	25	10	Pseudo-second-order model	Freundlich isotherm model	[127]

Note: “/” indicates no relevant data mentioned in the reference.

#### 3.2.2. Summary of Dye Removal

In summary, the adsorption of dyes on nanofiber membranes is mainly physical, with electrostatic attraction being the dominant force. This is because the pH of the solution affects the degree of ionization of the dye and the charge on the surface of the ENM. In our aforementioned work, hydrophilic groups were added to the surface of the ENM by modular assembly or chemical modification to increase the hydrophilicity of the ENM. The protonated amino groups exhibit strong electrostatic attraction to anionic dyes. This affinity improves the efficiency of dye adsorption. By increasing the number of functional groups (such as hydroxyl or carboxyl) [128,129], the prepared nanofiber membrane can absorb anionic and cationic dyes simultaneously, further expanding the practicality of the nanofiber membrane. According to the experiment of Fang-fang Ma et al. [52], the prepared PVDF@PDA@PPY composite nanofiber membranes can adsorb not only cationic dyes and anionic dyes but also adsorb heavy metal chromium ions. However, how to further improve the adsorption efficiency of using ENM in practical applications is a question we need to consider in the future.

### 3.3. Factors Affecting Performance

In addition to the pH of the solution affecting the nanofibrous membrane, the adsorbent loading also affects the adsorption of the nanofibrous membrane. For example, according to Katherine E. Greenstein et al., who prepared nanofiber membranes with α-Fe_2_O_3_ [78], it was found experimentally that as the content of α-Fe_2_O_3_ increased, the removal efficiency of the nanofiber membranes for heavy metal ions increased, mainly attributed to the increase in adsorption sites, resulting in more heavy metal ions being adsorbed onto the surface of the nanofiber membranes. In addition, the initial concentration of the solution affects the adsorption of heavy metal ions by the membrane. According to Zhang-Qi Feng et al. [54], the adsorption of chromium ions by the prepared porous PAN/GO nanofiber membranes gradually increased in capacity with increasing initial concentration, but as the adsorption sites saturated, the adsorption gradually reached saturation with increasing initial concentration of the solution. Coexisting examples also affect the adsorption of heavy metal ions by membranes [77]. Of the commonly co-occurring anions, only sulphate and nitrate reduced the removal of Cr(VI) by the nanofibers but did not affect the structure of the adsorbed Cr. Copper can compete with Cr(VI) for complexation with amine groups, thus attenuating the reduction of Cr(VI). In addition to this, factors such as contact time and temperature of the solution can also affect the adsorption of heavy metal ions by the nanofiber membrane.

The contact time of the dye also affects the adsorption of the dye by the nanofiber membrane. According to Fang-fang Ma et al. [22], the adsorption of MB by the prepared GO/PVDF increased with time, but the adsorption capacity of the adsorption gradually reached a plateau when the adsorption sites on the membrane surface reached saturation. Additionally, the loading amount affects the adsorption of dyes by nanofiber membranes. For example, the PVDF/PDA composite membrane [120] prepared by Fang-fang Ma et al. showed the best adsorption of MB at a loading concentration of 30 for PDA mainly because at too high a loading, too much loading on the membrane surface tends to collect on the surface of the membrane, resulting in a lower adsorption capacity. In addition to this, temperature, initial concentration, etc., also affect the adsorption of dyes by the membrane.

### 3.4. The More Advantageous ENMs

The high specific surface area and adjustable functionality of nanofiber membranes make them much more effective than conventional membranes for surface adsorption treatment of wastewater, with high porosity (typically around 80%) and fully interconnected pores with controlled pore size distribution from micron to submicron, which also makes them very suitable for a wide range of filtration applications [130,131]. The mechanisms of metal ion and dye removal using electrostatic spinning membrane technology mainly include physical adsorption and chemisorption.

Physical adsorption is mainly caused by the electrostatic or intermolecular forces between the adsorbent and the metal ions. For the adsorption of ionic pollutants, physical adsorption, and ion exchange adsorption methods are mainly used. Physical adsorption relies mainly on electrostatic and intermolecular forces between the adsorbate and the anion. Ion exchange relies on the adsorbent releasing ions while adsorbing the same number of ions, thus achieving the remobilization of the contaminant in the water. Common materials such as GO, polydopamine (PDA), β-cyclodextrin (β-CD), and magnetic nanoparticles have been widely used to remove metals and dyes from water [132,133,134].

Chemisorption is an effective method for the adsorption of nonionic dye contaminants. It relies mainly on the formation of strong chemical bonds or surface complexes with metals or dyes by functional groups on the adsorbent material. Polydopamine (PDA), polyvinyl fluoride (PVDF), GO [22,52,135], etc., can form complexes with metal ions and dyes through their special structural properties, thus increasing the adsorption capacity. 

In summary, the specific surface area and chelating ability of the adsorbent are the main determinants of physical and chemical adsorption, respectively; therefore, loading physical adsorbent materials onto the surface of ENM to effectively increase the specific surface area of the adsorbent material can achieve efficient adsorption. Compared with physical adsorption, chemisorption mainly relies on the formation of stable chemical bonds or redox reactions between adsorbent materials and ions to achieve water treatment, and according to this principle, excellent adsorbent materials can be obtained by grafting functional groups directly on the surface of spun-bond polymers. From the current research, among them, PDA and GO are used for ENMs to improve their adsorption performance, which is a more mature and effective method.

## 4. Summary and Outlook

Water pollution caused by heavy metals and dyes has become one of the major environmental problems of the 21st century. In wastewater treatment technology, ENMs have attracted great interest due to their good permeability, stability, easy handling, and low energy consumption [75,136,137]. In the past, innovative developments in electrospinning equipment and technology have enabled the functionalization of nanofibers for electrospray and multi-jet electrospinning [138]. At the same time, electrospinning technology is widely used in the preparation of ENMs due to their high porosity, ease of preparation, and high specific surface area [139]. Nanofiber membranes are also well used in industrial production for water treatment [11,140]. In this review, we report the latest research on the removal of heavy metals and dyes from water by nanocomposite fiber membranes. Furthermore, new materials and theories for the removal of heavy metal ions and dyes by ENM adsorption are analyzed, showing that these electrospun nanofiber membranes exhibit excellent performance in the treatment of heavy metal ions and dyes in water, proving their potential application in wastewater treatment.

Electrostatic spinning offers a simple and versatile method for the preparation of membranes for water treatment, and despite considerable progress, there is still a need to improve efficiency and flux rates by further optimizing the composition, structure, and physicochemical properties of nanofiber membranes, and future work should focus on a comprehensive understanding of the active adsorption sites exposed to the fiber surface, the mechanisms of desorption of ions/molecules, and the transport kinetics in the interconnected pores [141]. Despite these efforts, there are challenges to the further expansion and growth of ENM applications. Firstly, due to the scaling behavior of the ENM after cycling, the actual flux will be much lower than the theoretical value; secondly, in the presence of multiple metal ions or high flux, functionalized or deposited nanoparticles on the surface of the nanofiber membrane may be detached from the nanofibers, and this possible transformation alters the water flux and the adsorption capacity of the ENM for heavy metal ions and dyes, resulting in a reduced removal capacity; finally, the synergy between the membrane material and the surface roughness has to be considered, as it may also affect the adsorption capacity of the coating for dyes and heavy metal ions. While surface functionalization of membranes can significantly improve their filtration performance, it typically involves multiple steps, making it less suitable for industrial product development. It should be used to achieve large-scale production by further simplifying the single manufacturing process. In addition, as the manufacture of some fibrous membranes requires the use of hazardous organic solvents as solvents for the working fluid, further methods for the environmentally friendly post-treatment of fibrous membranes should be sought. Furthermore, to make full use of clean energy and reduce costs, new fiber membranes should be developed that can make full use of sunlight for rapid water treatment (e.g., decontamination, disinfection, and desalination) to directly produce drinking water with high throughput and low cost, which is particularly important for developing countries.

The key issue is that the practical application of ENMs in wastewater treatment needs to be further promoted; at present, these membranes have made great progress in laboratory-scale applications [142], but their mass production in industrial applications is still limited. Although there are still many problems with ENM, such as the need to further improve mechanical properties and production efficiency, it is hoped that in the future, these difficulties will eventually be overcome with the advancement of nanofiber technology and the increased requirements for water environmental management.

## Data Availability

The data presented in this study are available upon request from the corresponding author.
